# Real-time detection of loosening torque in bolted joints using piezoresistive pressure-sensitive layer based on multi-walled carbon nanotubes reinforced epoxy nanocomposites

**DOI:** 10.1038/s41598-025-89290-9

**Published:** 2025-02-09

**Authors:** Abdulkadir Sanli, Bilgehan Demirkale, Olfa Kanoun

**Affiliations:** 1 Chair for Electrical Measurements and Sensor Technology, Technische Universität Chemnitz, Reichenhainer Str. 70, 09126 Chemnitz, Germany; 2 Chair for Assembly and Handling Technology, Technische Universität Chemnitz, Reichenhainer Str. 70, 09126 Chemnitz, Germany; 3 Department of Bioengineering, Royal School of Mines, Imperial College London, London, SW7 2AZ UK

**Keywords:** Bolt-loosening, Smart washer, Multi-walled carbon nanotubes, Pressure sensing, Epoxy resin, Piezoresistivity, Structural health monitoring, Nanoscale materials, Structural materials, Mechanical engineering

## Abstract

Bolted connections are extensively used in construction and machine design, playing critical roles in various industrial applications. Bolts and screws are, however, prone to loosening or separating due to factors such as shock, vibration, and temperature fluctuations. This self-loosening phenomenon poses a considerable challenge to the reliability of bolted connections, necessitating regular inspections to ensure their safety. In this study, we report a cost-effective technique for monitoring bolted joint loosening torque: BoltWISE (Bolt loosening detection with innovative sensors). BoltWISE employs an innovative sensor element consisting of a piezoresistive, pressure-sensitive layer made from multi-walled carbon nanotube/epoxy nanocomposites coated onto an FR4 substrate, functioning as a washer. Our approach provides high sensitivity, durability, linearity, and fast response times, with minimal hysteresis during both the tightening and loosening processes. Finite-element method simulations were conducted to determine the optimal sensor positions, ensuring fast response during bolt tightening and loosening. Our findings highlight BoltWISE as a promising, low-cost solution for efficiently detecting bolt loosening in industrial environments. Its ease of implementation and fabrication make it a valuable tool for improving safety and maintenance practices across various industries.

## Introduction

Bolted joints are crucial for the safe operation of various types of equipment, and they are widely used in many mechanical and civil applications, including manufacturing, power generation, transportation, and mining^1^. They are easy to install and disassemble for maintenance, are cost-effective, and can bear relatively heavy loads^2^. These joints, however, tend to loosen over long periods, which can occur separately or in combinations of many factors. For instance, vibration can eventually cause the bolt to “unwind” from the mating threads and the joint to lose its clamp force. In addition, under environmental changes or cyclic industrial processes, loosening of the bolt can also occur due to differential thermal expansion of the material between the bolt and the joint. A sudden force applied to the bolt or the joint due to dynamic loads can cause a mechanical shock, loosening the bolts. If the bolt is not re-tightened in time, this loosening may lead to **serious** damage to equipment or even catastrophic accidents and disasters^3–5^. Hence, real-time monitoring of the state of bolted joints to prevent them from loosening is highly required.

To date, various techniques have been developed in mechanical and civil structures, as well as in medical dental implants, to observe the state of the bolt, namely whether the joint has been tightened to its full torque^6^. In civil structures, human visual inspection or torque wrench technique using torque meters has often been used for detecting bolt loosening. Here, educated bridge inspectors are appointed to detect and record various bridge structural defects, including bolt loosening. Despite its simplicity and cost-effectiveness, inspection results give many errors due to inconsistency in inspection skills and the limited abilities of the inspectors to identify the deficiency in bolts, and these approaches cannot monitor the state of the structures online^7^. Other methods for assessing bolt/nut assembly tightness include wave energy dissipation-based linear acoustic approaches^8,9^, contact acoustic nonlinearity-based vibroacoustic modulation^9,10^, and Hilbert Huang Transform (HHT) method-based detection^11,12^. These techniques often involve costly, frequent audits and labor-intensive processes. Additionally, ongoing research focuses on advanced bolt-loosening detection technologies that employ sensors and transducers, including piezoceramic-based transducers^13–15^. In this approach, a washer acts as a transducer by securing the piezoceramic patch between two pre-machined flat metal rings. This setup requires, however, additional costly components for the data acquisition. Conventional metallic strain gauges are used as washers or integrated bolts^16,17^. Even though this approach yields quite good accuracy, it is expensive and, therefore, can be applied only for experimental applications. Deep learning-based bolt loosening detection systems^18–20^, while at the forefront of innovation, face limitations due to their reliance on labeled training data, which may not capture the full spectrum of real-world variations and conditions. *In-situ* inspection methods, including visual inspection, such as image processing methods and smart monitoring techniques, i.e., acoustic, elastic wave, electromechanical impedance, magnet field, and RFID sensor tag, etc.^21–26^ have been studied. Sun et al.^27^ proposed a method for detecting bolt loosening in steel bridge joints via image processing, which captures images of bolt joints and calculates rotation angle changes. While vision-based methods can detect minor bolt rotations corresponding to small length variations, these methods often face challenges in practical applications due to factors such as lighting, camera resolution, and the need for complex image processing algorithms^28–30^. Moreover, such methods may require costly and sophisticated monitoring systems. In contrast, BoltWISE provides a more straightforward, cost-effective, and reliable solution for monitoring bolt-loosening events, making them more practical for widespread industrial use.

**Fig. 1:**
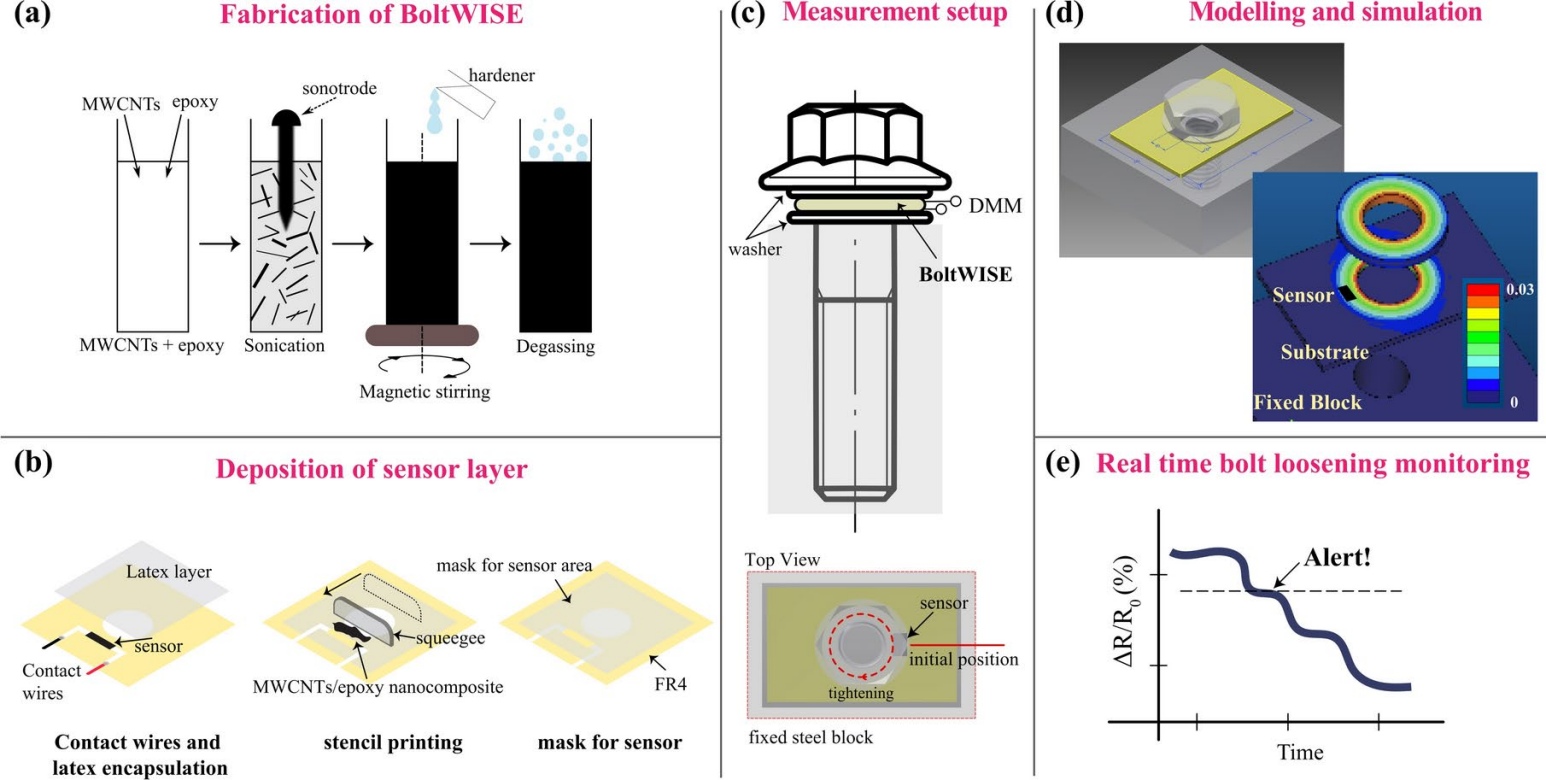
Fabrication and working principle of BoltWISE. (**a**) Schematic representation of the synthesis and (**b**) deposition of MWCNTs/epoxy nanocomposite layer on FR4 substrate. (**c**) Integration of the BoltWISE between two washers and top view of the measurement setup showing the location of the sensor. (**d**) Modeling and FEM simulation of the BoltWISE tightened in a fixed steel block under the load to define the most suitable location for the sensor. The 3D model was created using Creo (PTC Inc., Version 8.0, https://www.ptc.com/en/products/creo), and the finite element method (FEM) simulation was conducted using COMSOL Multiphysics (COMSOL Inc., Version 5.6, https://www.comsol.com/). (**e**) Real-time bolt torque loosening monitoring based on the relative resistance change; illustrations not to scale.

In this work, we report our real-time bolt loosening monitoring platform: BoltWISE (**Bolt** loosening detection **w**ith **i**nnovative **s**ensors), a simple and cost-effective technique for monitoring loosening torque on bolted joints. Unlike many existing methods that require complex and expensive instrumentation, BoltWISE offers a practical solution that can be scaled to various industrial applications. The system comprises a washer coated with a pressure-sensitive piezoresistive MWCNTs/epoxy nanocomposite layer (Fig. 1). Here, we deposited a pressure-sensitive layer on a glassy-epoxy FR4 substrate by a stencil printing technique, which is then located between the screw washer and nut. We have extensively characterized the pressure-sensitive sensor layer by investigating the dispersion state of the nanocomposite and developed a finite element model (FEM) to define the most appropriate location of the sensor by monitoring the amount of stress and stress distribution on the substrate and the sensor layer. Last, we studied the piezoresistive response of the sensor under different tightening and loosening torques to determine its sensitivity, stability, response time, and linearity. BoltWISE provides a streamlined, scalable, and efficient solution that is expected to reduce maintenance costs and prevent potential structural failures in critical industries such as aerospace, automotive, and civil infrastructure.

## Results and discussion

### Nanocomposite synthesis

The as-received MWCNTs from Southwest Nano Technology have a 95% purity, an outer diameter of 6–9 nm, and a length of < 1 µm. A concentration of 1 wt% MWCNTs is directly mixed with epoxy resin L20 (Bisphenol A-epichlorohydrin), and the mixture is then sonicated using a horn sonicator (Bandelin GM3200) for 30 min at 30 W power (Fig. 1a). The composite mixture is then mixed with a magnetic stirrer at 400 rpm for 2 h. Next, hardener (EPH-161), at a 1:10 ratio is added to the MWCNTs/epoxy mixture, and the solution is further mixed using a magnetic stirrer at 400 rpm for 10 min. The final mixture is put into a vacuum chamber to allow degassing that occurs during mixing processes.

### Sensor characterization

**Fig. 2:**
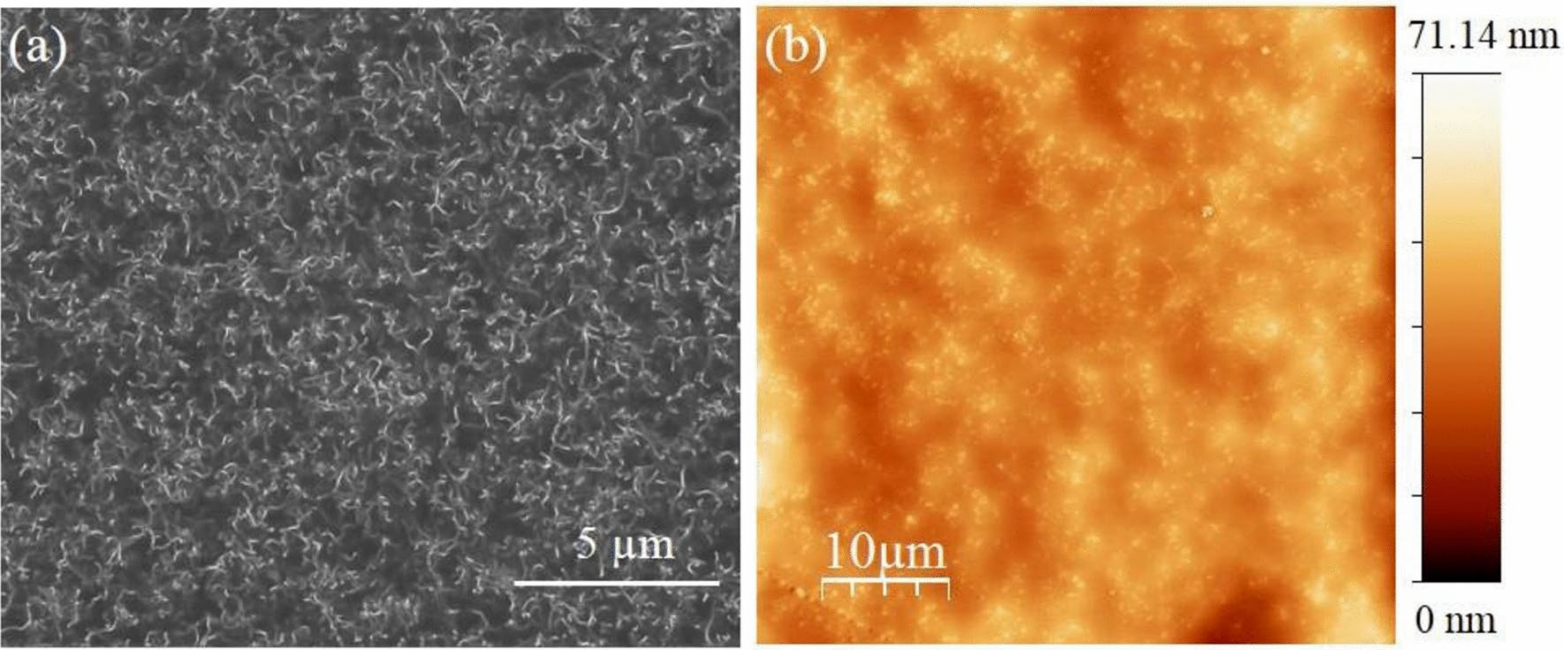
Surface morphology of the (MWCNTs)/epoxy-based pressure-sensitive nanocomposite with a 1 wt% MWCNTs concentration. In (**a**), scanning electron microscopy (SEM) was utilized to examine the surface structure, while in (**b**), atomic force microscopy (AFM) was employed for topographical analysis. Notably, the dimensions of AFM images are 50 µm by 50 µm, with a scale of 10 µm. The AFM image acquisition rate was set at one-line s^−1^. The analysis was conducted utilizing commercial WSxM SPM software.

The homogeneous distribution (dispersion) of MWCNTs within the epoxy matrix, as observed in the SEM image is crucial for ensuring a repeatable and sensitive sensor response^31,32^. Despite the presence of some partial agglomerations, the MWCNTs are found to be randomly and uniformly distributed (Fig. 2a). Furthermore, AFM topography (Fig. 2b) reveals a relatively smooth surface, with MWCNTs not visibly present on the surface, suggesting their burial within the matrix. Nevertheless, the quasi-homogeneous distribution of the MWCNTs network within the epoxy polymer matrix, confirmed by the AFM image, underscores the importance of achieving uniform dispersion for optimal sensor performance.

### Finite element modeling

Computational modeling and simulation are essential in developing the nanocomposite-based sensor system, providing insights into its mechanical behavior, stress distribution, and displacement, which help optimize design and improve measurement accuracy.

To ensure the safety of the measurement system, we built a 2-D FEM model to understand the stress and displacement over the sensor area and to determine the ideal sensor position during tightening. It is important to note that the forces generated by the tightening of the bolt cannot be defined using a single, standard force definition. Instead, they are structurally formed between the bolt and the toothed steel block (Fig. S2). To model this accurately, we relied on the force distribution principles for tensioned bolted joints, specifically the tension diagram of bolt connections in the assembly state^33^. This approach allowed us to capture the internal stresses (tension) and deformation behavior when bolts are tightened during installation. These forces were carefully considered in our FEM analysis to ensure accurate stress and displacement representation over the sensor area and to determine the ideal sensor position during tightening.

**Fig. 3:**
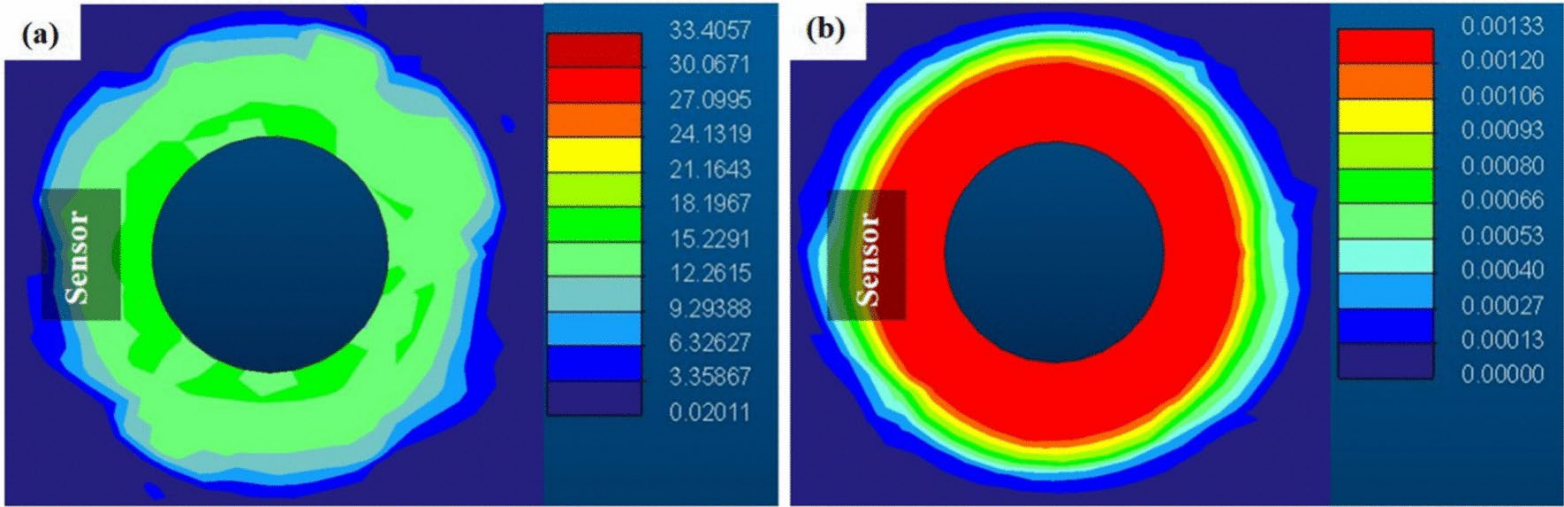
2-D FEM model of the bolt when the screw is tightened up to 10 Nm of torque. (**a**) The stress (in MPa) distribution across the bolt structure is depicted. The color gradient represents varying stress magnitudes, with warmer colors indicating higher stress levels and cooler colors indicating lower stress levels. The position of sensors within the bolt structure is marked, allowing for precise monitoring and analysis of stress variations at specific points along the length of the bolt. (**b**) The displacement distribution (in mm) of the substrate material under the applied tightening torque. Similar to the stress distribution visualization, the color gradient illustrates varying displacement magnitudes, with warmer colors representing larger displacements and cooler colors indicating minimal displacement.

We observed that the amount of stress is more dominant around the bolt, gradually decreasing as it gets away from the sensor area. Furthermore, it is calculated that under 10 Nm of torque, up to 21 MPa of stress (corresponding to ~ 31.5 kN) is being applied to the sensor (Fig. 3a). The substrate displacement from the bolt and sensor area is also calculated in Fig. 3b. As expected, the maximum displacement occurs around the inner area of the bolt when tightened and decreases towards the outer. Under the maximum torque of 10 Nm, the displacement on the sensor area is calculated to be in the range of 0.9–1.3 µm, which again confirms that the sensor was in the optimal position for monitoring the bolt loosening.

### Piezoresistive response of the pressure-sensitive nanocomposite layer

**Fig. 4:**
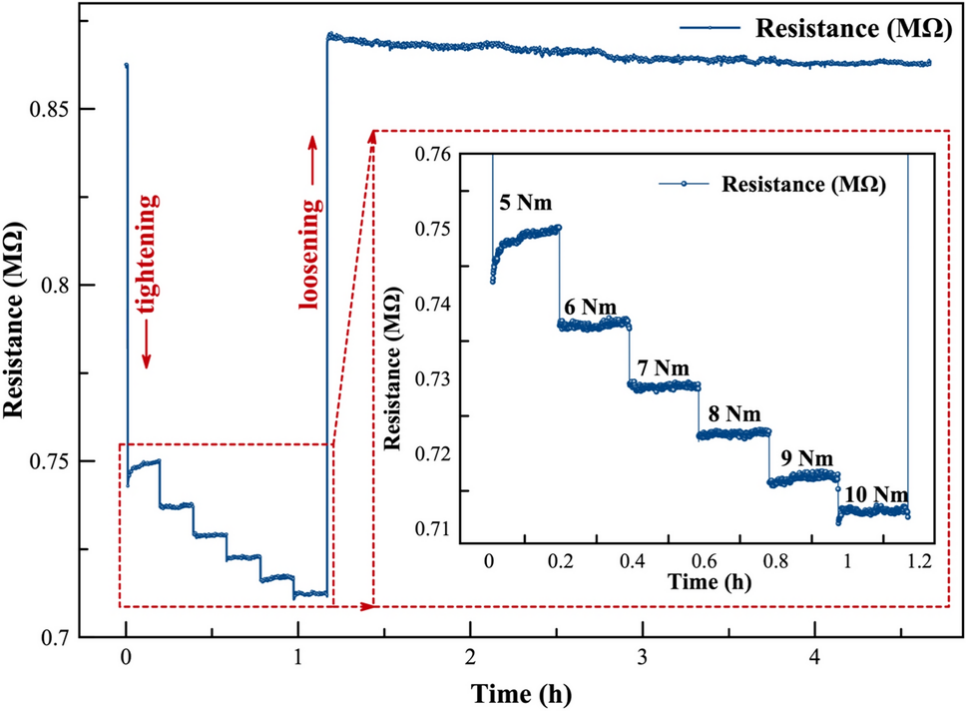
The resistance profile of the sensor after tightening and loosening the bolt. The inset figure displays the piezoresistive response of the sensor at various torque levels, ranging from 5 to 10 Nm, incrementing by 1 Nm torque. Throughout this experiment, measurements were taken continuously for 30 min at each torque step.

Figure 4 shows the resistance change of the MWCNTs/epoxy nanocomposites under tightening/loosening of the screw. We observed that the resistance of the sensor decreases with the tightening and increases with the loosening of the screw, with a quite sensitive response. With the application of the initial first 5 Nm torque, the sensor resistance decreases from 0.86 to 0.75 MΩ (around 12.8%), and it increases up to 0.71 MΩ under 10 Nm of the tightening torque.

The inset image (Fig. 4) shows that the sensor is also quite sensitive under the 1 Nm of tightening/loosening torque. Both experimental and numerical studies have demonstrated that the piezoresistivity mechanism in the MWCNTs/polymer nanocomposites under pressure can be attributed to three main factors: (i) changes in tunneling resistance within the CNTs network, (ii) breakage or loss of contacts between the CNTs in the polymer matrix, (iii) piezoresistivity of individual CNTs themselves^34–36^.

**Fig. 5:**
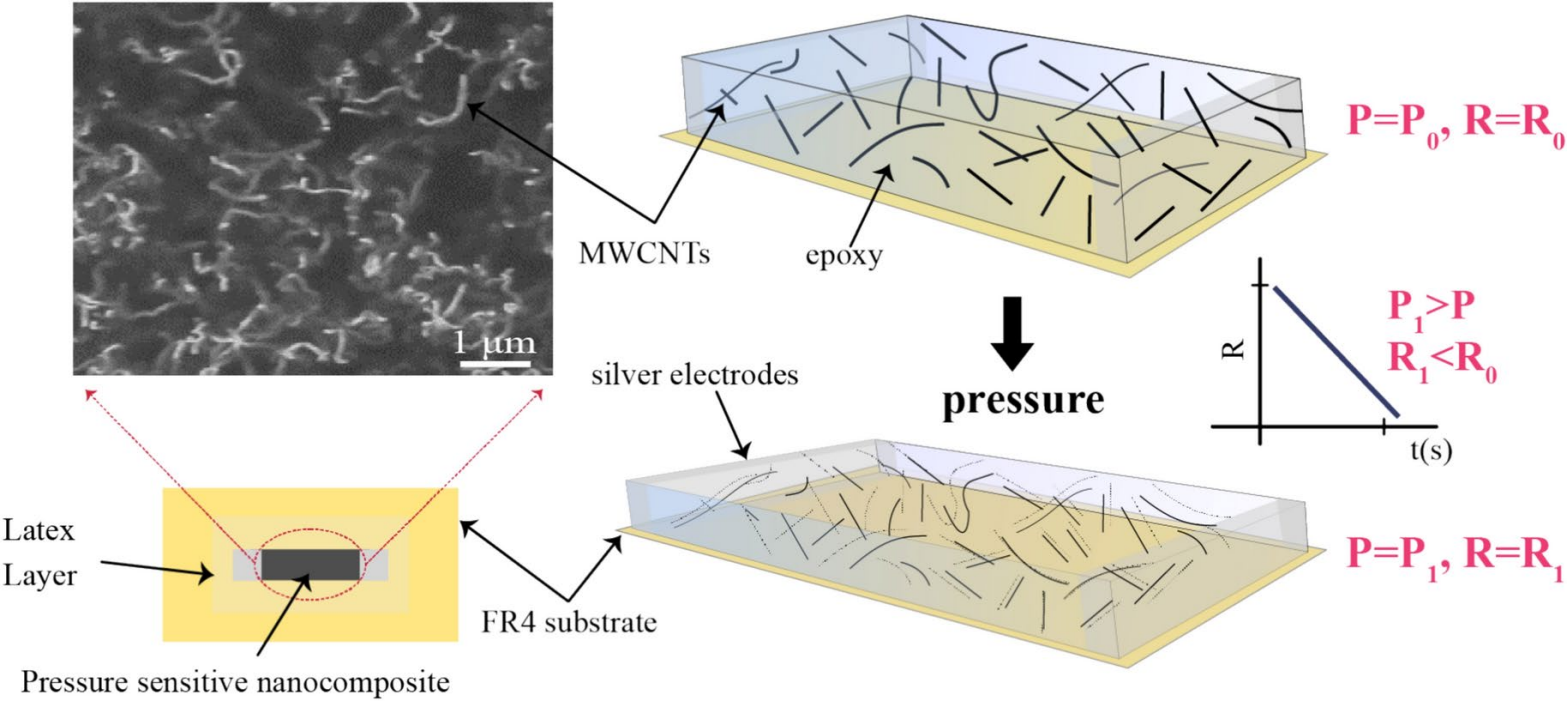
A schematic illustration of the piezoresistivity mechanism observed in MWCNTs/epoxy nanocomposites under applied force, accompanied by a corresponding SEM image showcasing the uniform dispersion of MWCNTs network within the epoxy resin polymer matrix. As pressure is exerted, the interconnected network of MWCNTs within the composite material responds dynamically by drawing closer to each other. This spatial rearrangement of the MWCNTs network leads to a notable reduction in electrical resistance across the applied pressure.

In the case of CNTs/polymer-based nanocomposites, the CNTs are separated from each other by a small distance due to the inevitable gap between CNTs arising from the polymer host. Hence, the tunneling effect dominates the conduction mechanism of the nanocomposite under load^37,38^. The tunneling resistance is *R*_*tunnel*_ ∝ *(d) exp (cd)*, where *c* is constant, and *d* is the distance between CNTs^39,40^ Considering the aforementioned effect, the decrease in the resistance of the MWCNTs/epoxy nanocomposite under pressure can be explained as follows (Fig. 5): as confirmed through SEM/AFM images, MWCNTs are randomly and homogeneously distributed within the epoxy polymer matrix, which gives a specific resistance value. Under the applied pressure, MWCNTs get closer to each other, reducing the distance between them and resulting in a certain decrease in the total resistance, which strongly depends on the concentration of MWCNTs.^41^.

**Fig. 6:**
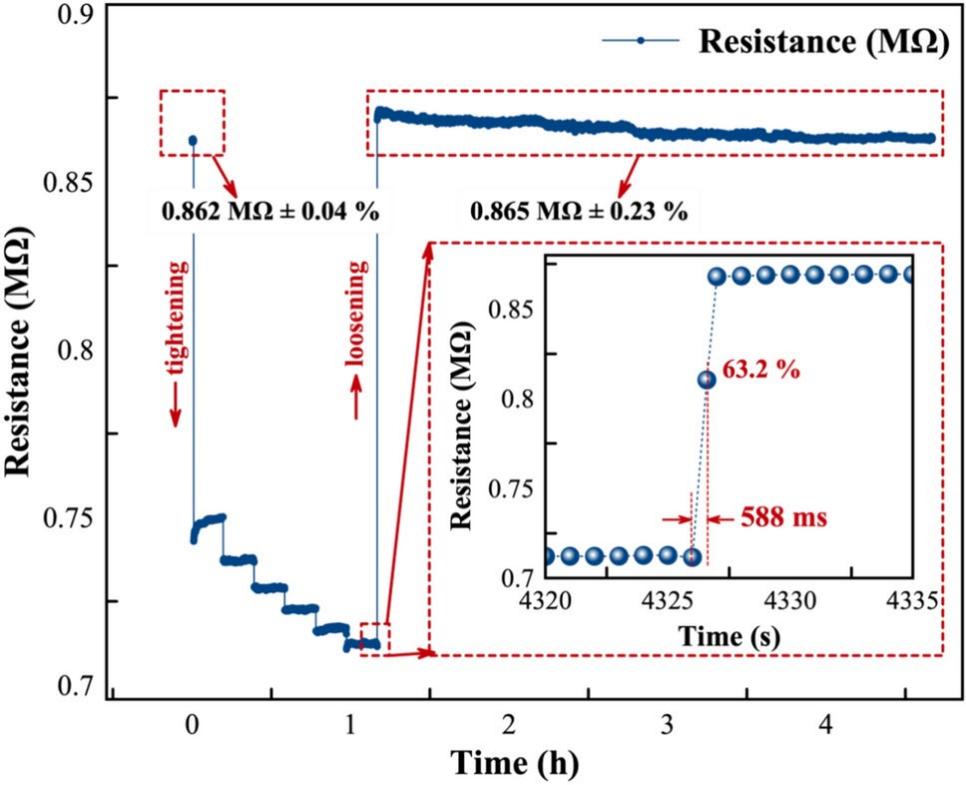
Initial and final resistance profiles of the sensor following the tightening and subsequent loosening of the screw. The inset figure provides further insight into the reaction time of the sensor during loosening. Remarkably, the sensor demonstrates an exceptional ability to recover rapidly to its initial position, indicating a swift response characteristic.

Besides the high sensitivity of the sensor under-tightening and loosening, the hysteresis and response time of the sensor are also important aspects to investigate^42^. Hysteresis in the nanocomposites occurs due to the viscoelastic nature of the polymer as well as the interaction between the fillers and polymers^43^. Depending on the strength of the interfacial binding between nanomaterial and polymers, CNTs/polymer-based nanocomposites give different hysteresis behaviors under load. While strong interfacial binding results in a lower hysteresis, weak interfacial binding results in a high hysteresis due to the high possibility of the slippage and irreversible rearrangement of CNTs^22,44^. The initial resistance of the sensor is 0.862 MΩ ± 0.04%, and it changes to 0.865 MΩ ± 0.04% after the tightening and loosening of the screw, which corresponds to a negligible relative resistance change of 0.3% that is attributed to the strong interfacial binding between MWCNTs and epoxy polymer matrix (Fig. 6).

We also studied the response time of the sensor (Fig. 6 inset). When the bolt starts to loosen, the pressure-sensitive layer reacts with a fast response of approximately 588 ms. The rapid reaction of MWCNTs/epoxy nanocomposites is directly linked to the fast re-organization of the MWCNTs network within the epoxy polymer matrix^45,46^. On the other hand, sensor linearity is a crucial parameter because it indicates the consistency of the response of a sensor across its specified measurement range. Depending on the type of application, the non-linear response could be a substantial disadvantage for the practical application of the sensor since non-linear responses require complex linearization methods, curve-fitting, and calibration^45–47^. The proposed sensor has quite a linear piezoresistivity response under applied tightening torque with a linearity factor of 0.96 (Fig. S4). The linear response of the sensor to varying torques is primarily due to the pressure-induced reorganization of the MWCNT network within the epoxy matrix. When torque is applied, the pressure-sensitive layer experiences mechanical stress, compressing the MWCNTs and increasing contact between neighboring nanotubes, which decreases the resistance. This leads to a measurable change in resistance, and the reorganization remains consistent across the torque range, resulting in a linear piezoresistive response. The linear response of the sensor confirms again that the proposed sensor can be easily used as a pressure sensor in the monitoring of the loosening of the screws in big machines in industrial plants.

## Conclusion

BoltWISE is a pressure-sensitive intelligent washer for monitoring the loosening/ tightening torques of the bolted joints. BoltWISE can be produced relatively low cost using the stencil printing technique with the materials for prototypes costing less than $1 in our lab (see Table S1). We expect this number to decrease substantially when the production is scaled up industrially. BoltWISE exhibited superior piezoresistive performance compared to existing solutions in terms of sensitivity, hysteresis, response time, and linearity. BoltWISE reported in this work, however, has at least four limitations:

(i) We used FR4 as a substrate because of its strong adhesion to the pressure-sensitive layer^48^. FR4, however, tends to fracture under high loads, which, as a result, can cause sensor failure. Using alternative materials, such as steel (with an insulating layer), could overcome this issue.
(ii) The sensor is sensitive to temperature and humidity^49,50^. These effects can be compensated by integrating a dummy sensor (not under load) placed close to the active sensor. The dummy sensor, which is not under load, would be exposed to the same environmental conditions (e.g., temperature and humidity), enabling us to subtract the environmental-induced resistance change from the load-induced resistance change. This approach would improve the accuracy of the preload monitoring system under varying environmental conditions.
(iii) The current sensor design is unsuitable for non-flat surfaces due to the limitations of the FR4 substrate; however, we have previously demonstrated that the MWCNTs/epoxy composite can also be deposited on a flexible polyamide substrate.^49,51^
(iv) Data transmission and monitoring of the current version of BoltWISE relies on wired connections and battery power for data transmission, which could limit its practical application. In future versions, using a mobile device, such as a mobile phone, the data generated by the sensors can be easily processed and stored on the cloud with remote access capabilities, essential for structural health monitoring applications.

BoltWISE demonstrates strong potential as a highly scalable smart washer for monitoring loosening torques in bolted joints of large industrial machines. It offers low service costs and improved sensor performance. While we have focused on loosening torques of the bolted joints in this work, this technology is suitable for a wide range of applications, including anterior cruciate ligament (ACL) surgery and dental implants.

## Experimental methods

### Deposition of nanocomposite layer and final sensor design

Due to its good mechanical properties and strong adhesion with the nanocomposites, a glass-reinforced epoxy substrate (FR4) is used for thin nanocomposite-based sensor layers^52^. After the dispersion preparation, the FR4 substrate is first covered with a mask for electrode deposition to perform the electrical measurements. Here, a certain amount of silver paste is applied on both sides manually and dried for 30 min at room temperature in a clean chamber to ensure good electrical contact between the nanocomposite layer and the silver paste. Then, a second mask with pre-defined patterns of 3 mm × 5 mm in size is applied on the substrate for nanocomposite deposition.

Due to the high viscosity of MWCNTs/epoxy nanocomposite, it is highly challenging to obtain a thin layer. We therefore used a stencil printing technique to deposit MWCNTs/epoxy nanocomposite on the FR4 substrate (Fig. S1). After the deposition of the nanocomposite layer and removal of the masks, the sample is dried for 3 h at 160 °C in a climate chamber. Finally, to prevent sensor damage during the load and minimize the humidity influences, a thin layer of latex polymer is applied onto the sensor and dried overnight.

### Determination of optimum sensor position and measurement setup

MWCNTs/epoxy nanocomposites-based force sensitive sensor layer is deposited on the FR4 substrate that is located between the washer and steel block (Fig. S3). Through a choice of a proper washer, the sensor area is covered to make sure that the pressure is being applied to the sensor layer.

In this work, we used a screw with the thread diameter of 10 mm (M10) and strength class of 8.8. According to the datasheet (DIN 13-1), the maximum torque that can be applied to the screw is 33.5 Nm. Since we aim to monitor bolt loosening and protect the sensor from damage, we applied a maximum of 10 Nm of torque to the sensor. A highly precise commercial torque wrench (Neutral, Würth Elektronik) is used to monitor the response of the sensor under applied pressure. The screw is tightened from 5 to 10 Nm with the torque step of 1 Nm, and the corresponding resistance values of the sensor are recorded by a custom-made LabVIEW GUI. For the stability test of the sensor under constant torque, the resistance value of the sensor is recorded over 30 min for each torque step.

### Microstructural characterization

SEM images were acquired using a Nova nanoSEM scanning electron microscope in high vacuum and through the lens detector with immersion mode. To monitor surface topology, Keysight Atomic Force Microscopy (AFM) is used. For imaging, conductive Cr/Pt-coated probes with a diameter of 10 nm, a resonant frequency of 75 kHz, and a force of 3 N/m are used.

### Computational modeling of bolt-loosening monitoring system

We used the commercial ANSYS Finite Element (FE) code to conduct FEM investigations. The model used in the analysis consists of an M10 screw, DIN 125-A washer (outer diameter 20 mm, inner diameter 10.5 mm), FR4 substrate, and a toothed steel block. FEM analysis is performed by the Fasteners module in the Simulation Tool of the PTC Creo© program.

## Supplementary Information

Supplementary Information.

## Data Availability

The datasets generated during and/or analyzed during the current study are available from the corresponding author upon reasonable request.
