# Supplementary material for: Real-time detection of loosening torque in bolted joints using piezoresistive pressure-sensitive layer based on multi-walled carbon nanotubes reinforced epoxy nanocomposites
Source: Sci Rep. 2025 Feb 9;15:4830. doi: 10.1038/s41598-025-89290-9 (PMC11808053; doi:10.1038/s41598-025-89290-9)
Supplement: Supplementary file 1 — Supplementary Information. [file 41598_2025_89290_MOESM1_ESM.docx]

**Supplementary Information** for

**Real-time detection of loosening torque in bolted joints using piezoresistive pressure-sensitive layer based on multi-walled carbon nanotubes reinforced epoxy nanocomposites**

Abdulkadir Sanli^1,3*^, Bilgehan Demirkale^2^ and Olfa Kanoun^3^

^1^Chair for Electrical Measurements and Sensor Technology, Technische Universität Chemnitz, Reichenhainer Str. 70, 09126 Chemnitz, Germany

^2^Chair for Assembly and Handling Technology, Technische Universität Chemnitz, Reichenhainer Str. 70, 09126 Chemnitz, Germany

^3^Department of Bioengineering, Royal School of Mines, Imperial College London, SW7 2AZ, London, UK

*Corresponding author: [a.sanli@imperial.ac.uk](mailto:a.sanli@imperial.ac.uk)

**Supplementary Figures**


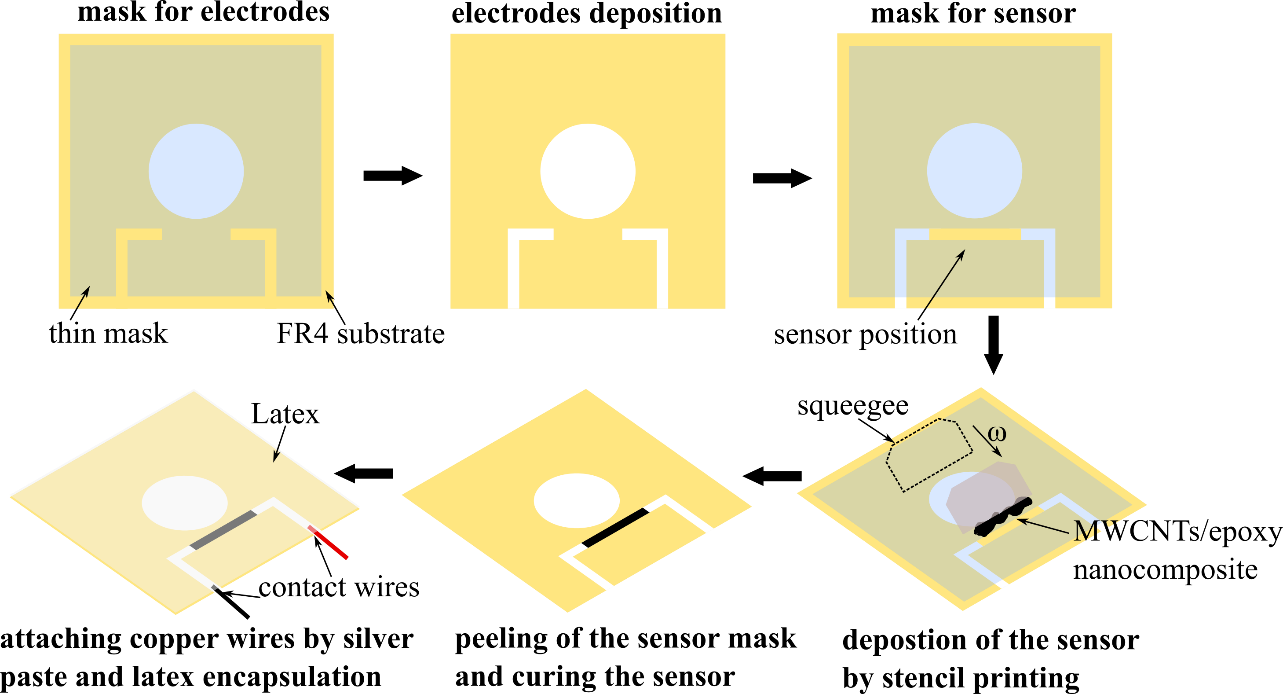


**Figure S1**: Schematic steps of the fabrication process of pressure-sensitive multi-walled carbon nanotubes (MWCNTs)/epoxy nanocomposite masks and electrode design, which are subsequently deposited onto a glassy-epoxy FR4 substrate. The process begins with the selection and preparation of the MWCNTs, followed by their dispersion within an epoxy matrix to form a homogeneous nanocomposite material. Next, the nanocomposite is deposited onto the FR4 substrate using a suitable technique, such as spin coating or screen printing, to create the desired mask and electrode patterns. These patterns are carefully designed to facilitate the sensing of pressure variations. The deposition process is followed by curing to ensure the adhesion and stability of the nanocomposite on the substrate. The resulting structure exhibits pressure-sensitive properties due to the unique conductivity changes induced by mechanical deformation of the MWCNTs within the epoxy matrix.


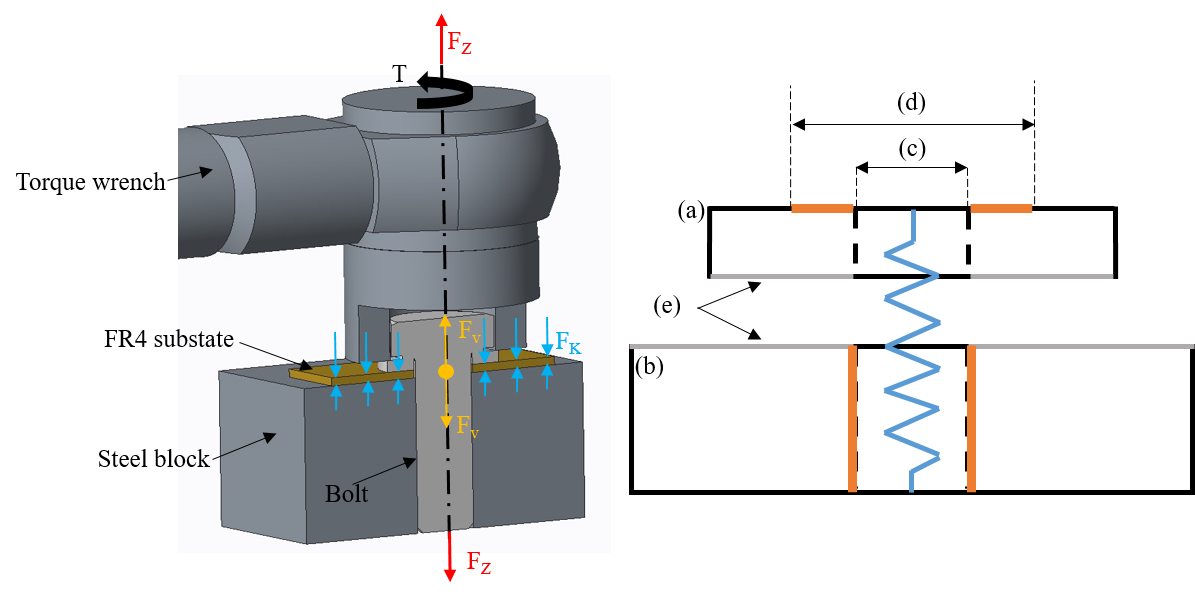


**Figure S2**: Cross-sectional view of the bolt and the forces caused by tightening the torque wrench. *F_v_*: Preload Force, *F_z_*: Tension Force, *F_k_*: clamping force and FEM analysis model of the bolt connection (a) FR4 substrate, (b) toothed steel block, (c) bolt diameter, (d) fastener head, washer diameter and (e) contact interface occurring between the sensor substrate/sensor and toothed steel block. The 3D model was created using Creo (PTC Inc., Version 8.0, https://www.ptc.com/en/products/creo),


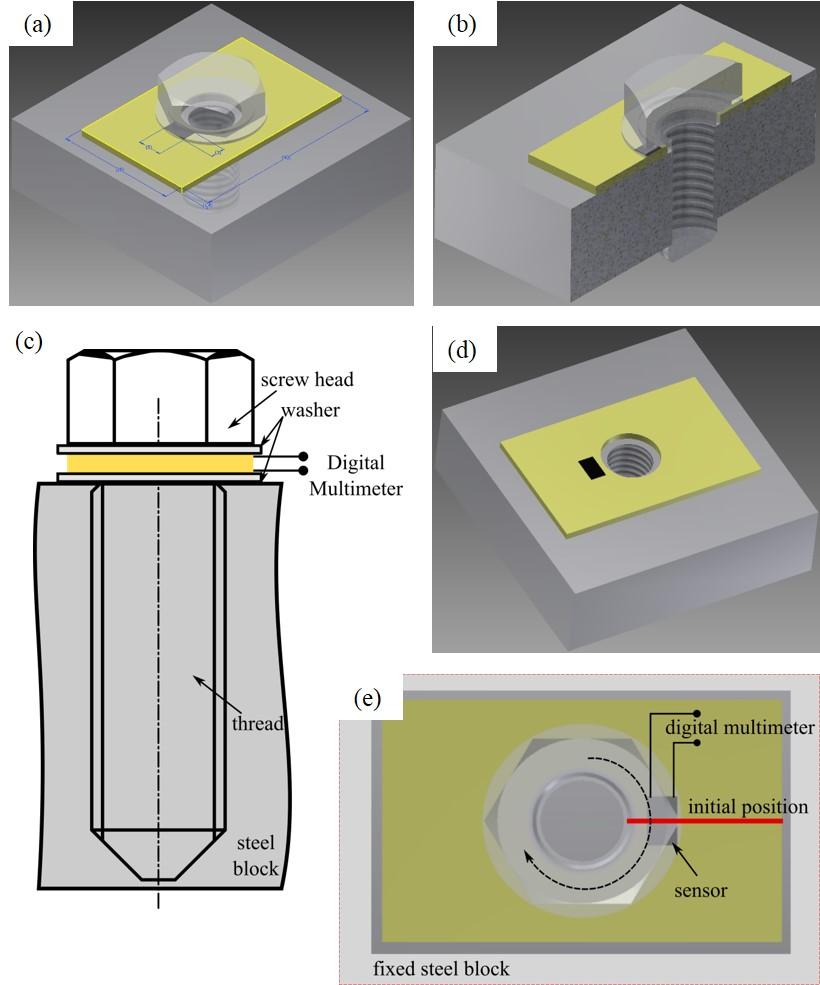


**Figure S3**: Measurement setup for bolt-loosening monitoring, (a) top-view (b) side-view showing that screw washer covers the entire area of the sensor, (c) side-view of the measurement setup showing that screw is in hard fixed toothed steel block, (d) position of the sensor and (e) measurement setup. The sensor dimension is 5 mm x 3 mm (length x height), and the chosen thread dimension and property class are 10 mm and 8.8, respectively. The 3D model was created using Creo (PTC Inc., Version 8.0, https://www.ptc.com/en/products/creo).


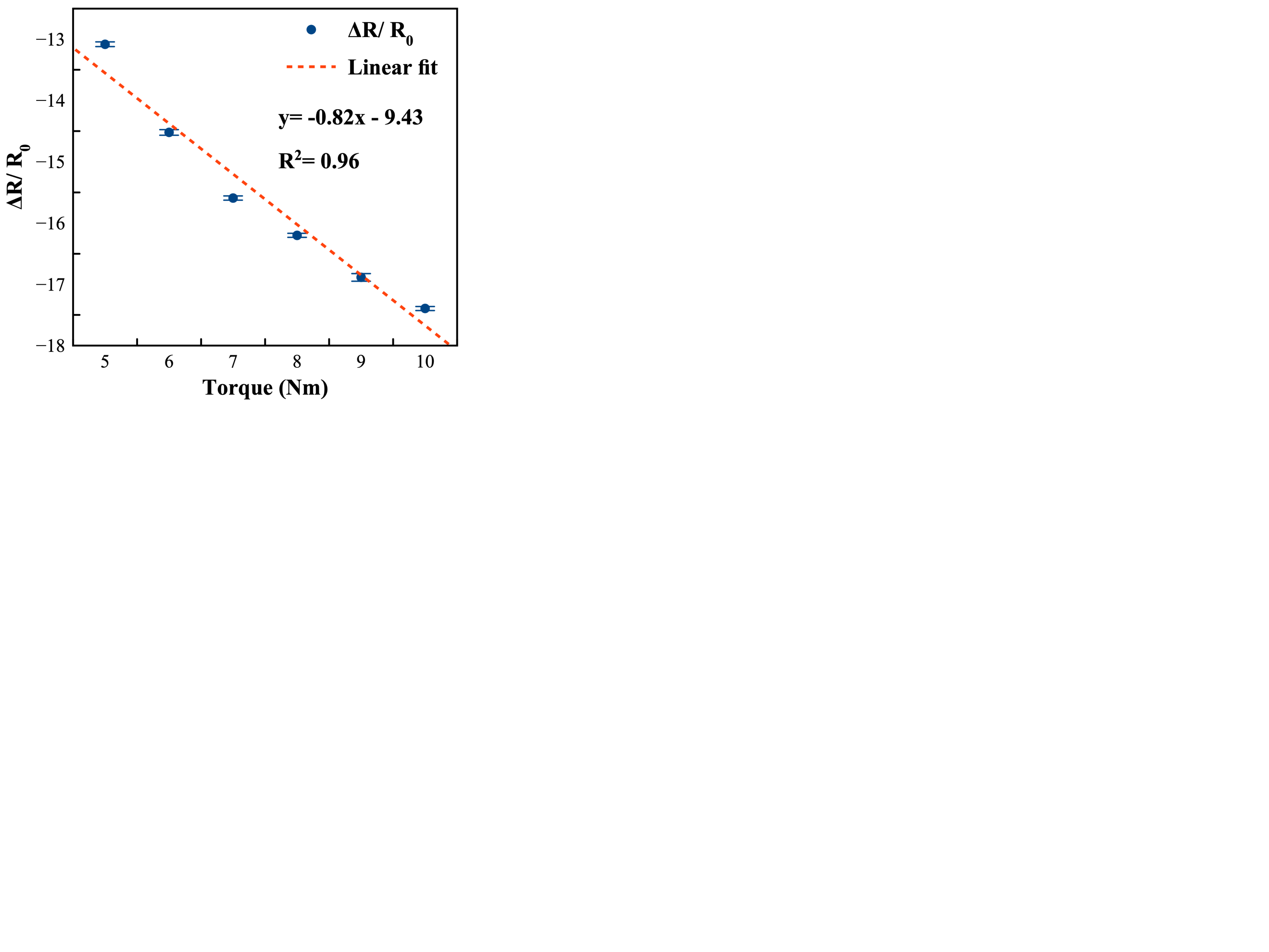


**Figure S4**: Linearity of the sensor under the applied torque, accompanied by the corresponding linear fit. We applied torque ranging from 5 Nm to 10 Nm with the step of 1Nm during the experiment. The linearity factor, calculated to be 0.96, indicates a high correlation between the torque and the response of the sensor (n=3).

**Table S1** – Cost of materials for one fully assembled BoltWISE platform.

| **Material** | **Unit** | **Price per unit** | **The amount used per sensor** | **Cost per sensor (USD)** |
| --- | --- | --- | --- | --- |
| **FR4 Substrate (3 x 5 mm)** | 3 x 5 mm piece | $27 (for 152 x 228 mm) | 1 piece (3 x 5 mm) | $0.53 |
| **MWCNTs** | 25 grams | $424 | 0.1 mg | $0.017 |
| **Washers (M10 pack of 100)** | 100 washers per pack | $7.5 | 1 washer | $0.075 |
| **Set Screws M10 x 25mm** | 100 screws per pack | $17.5 | 1 screw | $0.175 |
|  | | | **Total** | **$0.797** |

*Prices are converted from EUR using the prevailing exchange rate, subject to change.
